# Pocket to concavity: a tool for the refinement of protein–ligand binding site shape from alpha spheres

**DOI:** 10.1093/bioinformatics/btad212

**Published:** 2023-04-22

**Authors:** Genki Kudo, Takumi Hirao, Ryunosuke Yoshino, Yasuteru Shigeta, Takatsugu Hirokawa

**Affiliations:** Physics Department, Graduate School of Pure and Applied Sciences, University of Tsukuba, Tsukuba, Ibaraki 305-8571, Japan; Master’s Program in Medical Sciences, Graduate School of Comprehensive Human Sciences, University of Tsukuba, Tsukuba, Ibaraki 305-8575, Japan; Division of Biomedical Science, Faculty of Medicine, University of Tsukuba, Tsukuba, Ibaraki 305-8575, Japan; Division of Biomedical Science, Faculty of Medicine, University of Tsukuba, Tsukuba, Ibaraki 305-8575, Japan; Transborder Medical Research Center, University of Tsukuba, Tsukuba, Ibaraki 305-8575, Japan; Center for Computational Sciences, University of Tsukuba, Tsukuba, Ibaraki 305-8577, Japan; Division of Biomedical Science, Faculty of Medicine, University of Tsukuba, Tsukuba, Ibaraki 305-8575, Japan; Transborder Medical Research Center, University of Tsukuba, Tsukuba, Ibaraki 305-8575, Japan

## Abstract

**Summary:**

Understanding the binding site of the target protein is essential for rational drug design. Pocket detection software predicts the ligand binding site of the target protein; however, the predicted protein pockets are often excessively estimated in comparison with the actual volume of the bound ligands. This study proposes a refinement tool for the pockets predicted by an alpha sphere-based approach, Pocket to Concavity (P2C). P2C is divided into two modes: Ligand-Free (LF) and Ligand-Bound (LB) modes. The LF mode provides the shape of the deep and druggable concavity where the core scaffold can bind. The LB mode searches the deep concavity around the bound ligand. Thus, P2C is useful for identifying and designing desirable compounds in Structure-Based Drug Design (SBDD).

**Availability and implementation:**

Pocket to Concavity is freely available at https://github.com/genki-kudo/Pocket-to-Concavity. This tool is implemented in Python3 and Fpocket2.

## Graphical Abstract

**Figure:**
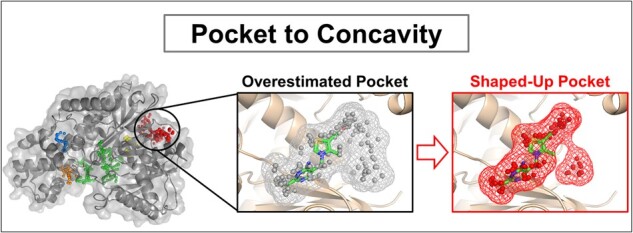

## 1 Introduction

Structure-based drug design (SBDD) is a method of designing drugs based on the structural information of their target proteins (Lionta et al. 2014). Recently, SBDD has been used often due to the increased number of available three-dimensional (3D) protein structures owing to crystal structure analysis and Cryo-electron microscopy (Renaud et al. 2018). Furthermore, predicted protein structures using AlphaFold2 accelerate SBDD (Jumper et al. 2021). The first step in SBDD is identifying the binding sites in the target protein structure. A pocket detection software is expected to provide information on the pockets, such as druggable sites, docking space, and desirable ligand size for drug discovery. Pocket detection software using an alpha sphere-based approach, such as Fpocket and Sitefinder on MOE (Chemical Computing Group Inc.), generates pseudo-atoms (alpha-spheres) based on Voronoi tessellation of the protein surface and accurately predicts druggable sites and docking spaces (Le Guilloux et al. 2009; Schmidtke et al. 2010). However, it provides insufficient information on the desirable ligand size as the predicted pockets by pocket detection software are often larger than the volume of the actual bound ligands (Supplementary Table S1; Gagliardi et al. 2022). The lack of information causes a hindrance to accurate and rational drug design.

This study proposes a tool, Pocket to Concavity (P2C), that refines the pocket shape overestimated by the alpha sphere-based approach to follow the actual ligand shape. This tool helps understand binding sites more clearly to select deep concavity where the core scaffold can bind. The algorithm of P2C is simple and easy to implement, making it an effective and practical approach for accurate pocket refinement. P2C showed that ∼80% of the test dataset improved in the volume overlap between the actual bound ligand and the pocket.

## 2 Features of the P2C process

**Figure 1.**
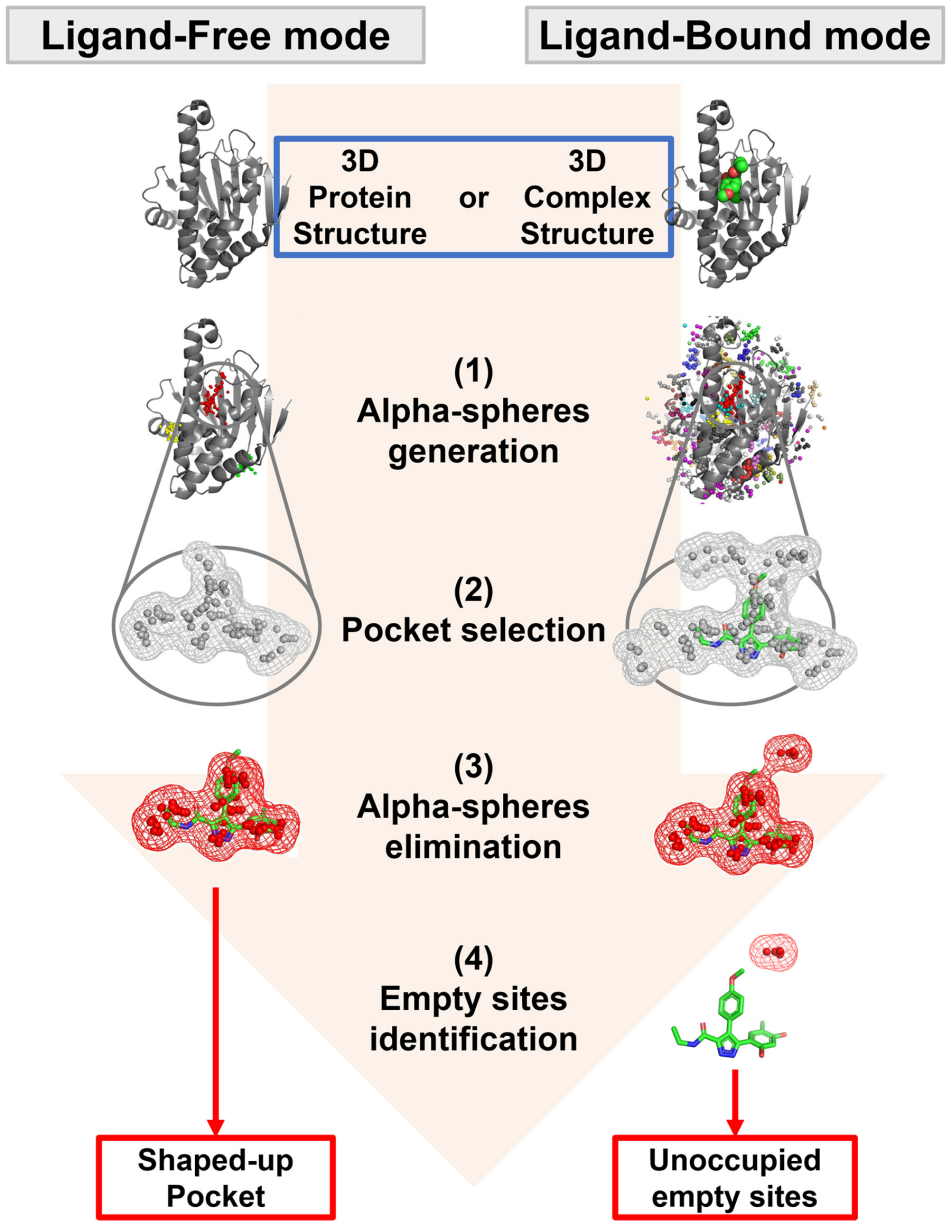
Pocket to concavity process. The input and output are shown in blue and red squares, respectively.

Figure 1 shows the workflow for P2C. There are two main modes: Ligand-Free (LF) and Ligand-Bound (LB) modes. The LF mode provides the shaped-up pocket using the 3D protein structure. Whereas the LB mode searches unoccupied empty pockets around the bound ligand using the 3D complex structure. Details of inputs and outputs are provided in the Supplementary Information (Appendix S0). The P2C process can be explained in the following four major steps (further information in Supplementary Appendix S1):

1. Alpha-spheres generation: The pockets and element, termed alpha-spheres, were generated based on Voronoi tessellation (Liang et al. 1998). Fpocket was used as the generator in the default P2C settings. The alpha-spheres coordinate file can be specified in place of this default generator.
2. Pocket selection: In the LF mode, pockets predicted by fpocket with high druggability were selected for P2C processing. In the LB mode, pockets around the bound ligand in the complex structure were selected for P2C processing. The range of alpha-spheres around the ligand can be specified.
3. Alpha-spheres elimination: Selected pockets were subjected to alpha-spheres elimination, the main step of P2C processing, and were refined for precise shape. In the elimination process, the density of each alpha-sphere was calculated, and alpha-spheres with low density were deleted (density parameter optimization is provided in Supplementary Information Appendix S2).
4. Empty site identification (the LB mode only): The refined pocket was aligned with the ligand, and the alpha spheres that overlapped the ligand were deleted. The remaining alpha spheres were re-clustered and re-defined as empty sites.

P2C was applied to the test dataset and Hit-to-Lead complexes. Approximately 80% of the complexes in the test dataset showed improved accuracy of the volume overlap between the refined pocket shape by P2C processing and the actual ligand. Furthermore, the alpha-spheres of complexes with hit compounds reproduced the shape of the lead compounds after P2C processing (Supplementary Appendixes S3 and S4). In addition, a case study for each mode is included in the Supplementary Information (Supplementary Appendix S5). The results suggest that P2C detects the deep concavity where the active ligand can bind. This tool is useful for identifying and designing desirable compounds in SBDD.

## 3 Conclusions

P2C can clearly and accurately refine pockets predicted by an alpha sphere-based approach. Refined pockets reproduce the deep concavity where the active ligand can bind. The algorithm is simple but effective and can be applied to various protein structures with or without the bound ligand. Using the 3D structure of the target protein, anyone can accurately predict the binding site and the shape of the actual bound ligand.

## Supplementary Material

btad212_Supplementary_Data
