# Supplementary material for: Pocket to concavity: a tool for the refinement of protein–ligand binding site shape from alpha spheres
Source: Bioinformatics. 2023 Apr 22;39(4):btad212. doi: 10.1093/bioinformatics/btad212 (PMC10148677; doi:10.1093/bioinformatics/btad212)
Supplement: btad212_Supplementary_Data [file btad212_supplementary_data.docx]

[Supplementary Information]

**Pocket to Concavity: A Tool for the Refinement of Protein-Ligand Binding Site Shape from Alpha Spheres**

Genki Kudo^1,*^, Takumi Hirao^2,3^, Ryunosuke Yoshino^3,4^, Yasuteru Shigeta^5^ and Takatsugu Hirokawa^3,4,*^

^1^Physics Department, Graduate School of Pure and Applied Sciences, University of Tsukuba, 1-1-1 Tennodai, Tsukuba, Ibaraki, 305-8571, Japan.

^2^Master's Program in Medical Sciences, Graduate School of Comprehensive Human Sciences, University of Tsukuba, Tsukuba, Ibaraki, 305-8575, Japan.

^3^Division of Biomedical Science, Faculty of Medicine, University of Tsukuba, 1-1-1 Tennodai, Tsukuba, Ibaraki, 305-8575, Japan.

^4^Transborder Medical Research Center, University of Tsukuba, 1-1-1 Tennodai, Tsukuba, Ibaraki, 305-8575, Japan.

^5^Center for Computational Sciences, University of Tsukuba, 1-1-1 Tennodai, Tsukuba, Ibaraki, 305-8577, Japan.

**Appendix 0: Input and output in Pocket to Concavity**

Pocket to Concavity (P2C) has two main modes, Ligand-Free (LF) and Ligand-Bound (LB). LF mode works with a Protein Data Bank (PDB) file of a three-dimensional (3D) protein structure provided by the user. LB mode requires an additional PDB file of a 3D ligand structure in the complex.

The default output files are stored in the “asphere_output” and the “p2c_output” directories in the execution path. “asphere_output” contains the results of the alpha-spheres generation. In addition, the alpha-spheres file generated by other pocket detection software can be used (note that parameter re-optimization is required for other generator conditions). “p2c_output” contains the P2C results.

**Appendix 1: Details of the P2C approach**

The P2C approach is executed as follows:

1. Alpha-spheres generation: First, alpha-spheres (pocket components) are generated using pocket detection software based on Voronoi tessellation. The default P2C approach uses fpocket as the default generator (le Guilloux *et al.*, 2009). The default generation generates alpha-spheres and binding pockets (alpha-sphere clusters) based on the 3D protein structure. In the LF mode, fpocket deletes tiny pockets (if the number of elements comprising the pocket < 30). On the other hand, the LB mode does not delete tiny pockets (in the fpocket command: -i 0). Other options are set to default in fpocket2 for the two modes. This step is not required if the alpha-spheres coordinate file is based on other software or if there are user settings.
2. Pocket selection: Among the pockets generated by the default generator, target pockets are selected to be analyzed by P2C. LF mode selects up to Xth pockets with the highest druggability as the target pockets. In LB mode, pockets within Y Å of the ligand are selected as the target pockets. The user can determine the X and Y values (default values are set to 1.0 and 1.7, respectively). This step is not required if there is an alpha-spheres coordinate file.
3. Alpha-spheres elimination: The selected pockets are refined to make their shape more proper and accurate. The density of each alpha sphere in the selected pocket is calculated, and is presented as the number of other alpha spheres within 1.53 Å from its center. The distance of 1.53 Å is adopted from the Csp^3^-Csp^3^ bond length, adjusting the scale to the binding distance of ligand atoms (Wunberg *et al.*, 2006; Su *et al.*, 2019). Subsequently, alpha spheres with a density value < 4 are removed (the cutoff “4” is optimized using the training dataset, shown in Appendix 2). Finally, the shaped-up pocket with high-density alpha-spheres is generated. The parameter must be re-optimized if the input alpha spheres are generated with other conditions.
4. Empty sites identification (LB mode only): Each shaped-up pocket and known ligand are represented by a 3D grid (spacing 1.0 Å), and shape comparisons are made between the pocket and ligand. Grids of the pocket overlapping with the ligand are deleted, and alpha spheres represented by the remaining grids are re-clustered. Pockets, after clustering, are re-defined as empty sites.

**Appendix 2: Parameter optimization**

In the alpha spheres elimination step of P2C, alpha spheres with low density were eliminated based on the cutoff, which was optimized using the training dataset. The training dataset was selected from protein-ligand complexes where the ligand was active enough to act as a drug. Specifically, 117 protein-ligand complexes with a -log (Ka) value greater than 7.0 in the CASF-2016 core set were selected for the training dataset (Table S1; Wunberg *et al.*, 2006; Su *et al.*, 2019). Each complex of the training dataset was downloaded from the PDB site (Berman *et al.*, 2000). Then, the complex and ligand structure files were prepared for each complex. Note that all hydrogen atoms in the ligand were removed, and the ligand was unified into heavy atoms only.

The training dataset was used to perform alpha-spheres generation, pocket selection, and alpha-spheres elimination. Options for the alpha-spheres generation were the same in the LB mode. In pocket selection, pockets within 1.7 Å of the ligand were selected as the target pockets. The distance of 1.7 Å was set to evaluate the shape comparison between the pocket and ligand. For the selected pockets, alpha-spheres elimination was performed separately under each condition, with the density cutoff ranging from 1 to 10. The smaller the cutoff is, the more lenient the process becomes, and the larger the cutoff is, the more severe the process becomes.

Next, we optimized the density cutoff that would give the best accuracy. The result with each cutoff was evaluated by Discretized Volume Overlap (DVO) in the optimization process. DVO has been used to evaluate pocket detection in DeepPocket by Aggarwal *et al.* (2021). Although they used DVO as the criteria for shape comparisons between the predicted pocket and actual pocket, this study used it for shape comparisons between the trimmed results of the pocket and ligand. In the DVO calculation, shape comparisons were made using the ligand and pocket grid points. 3D grid points with 1.0 Å spacing were generated on the complex structure. The grids closest to the ligand atomic center or alpha spheres center were labeled “ligand” or “pocket,” respectively. Concomitantly, the same label was placed on the grid points next to them. These grid points were used to assess the shape of the ligand and pocket. DVO was calculated using the following equation (1):

$\mathrm{DVO}=\frac{G_{\mathrm{ligand}}\cap G_{\mathrm{pocket}}}{G_{\mathrm{ligand}}\cup G_{\mathrm{pocket}}}$ (1)

where $G_{ligand}$ denotes grid points labeled “ligand” and $G_{pocket}$ denotes grid points labeled “pocket.” DVO is consistent with the Jaccard or Tanimoto index. Therefore, increased similarity in shapes between the ligand and pocket indicates that DVO is closer to 1, and the more different these shapes are, the closer the value is to 0.

This study performed the DVO calculation for each cutoff of the complexes (Table S1). The bar chart of the average DVO of 117 complexes in the training dataset is shown in Figure S1a. The horizontal line denotes the cutoff, and the default denotes the DVO between the ligand and pocket before the alpha spheres elimination. The average DVO before elimination (default in Figure S1a) was 0.306. For the cutoff ranging from 1 to 4, the average DVO for each cutoff was higher than that of the default. This result indicates that eliminating alpha spheres using these cutoffs can trim only those that do not bind to the ligand. In particular, cutoff 4 showed the highest DVO of 0.328. Contrastingly, when the cutoff was higher than 6, the DVO value was lower than the default. As a result, alpha spheres elimination with higher cutoffs was too severe to delete even the alpha spheres overlapping with the ligand position.

Furthermore, the cutoffs with maximum DVO for each complex of the training dataset were analyzed. Figure S1b shows the histograms with the most improved DVO complexes cumulated for each cutoff. At cutoffs 3 to 5, alpha-spheres elimination had more complexes with maximum DVO. Notably, the elimination with cutoff 4 had 23 complexes with maximum DVO. In addition, regardless of the cutoff, the DVO after elimination was higher than before for 90.6% of the training dataset. This result suggests alpha-spheres elimination could form clear ligand-binding sites in most complexes with the best cutoff at 4.


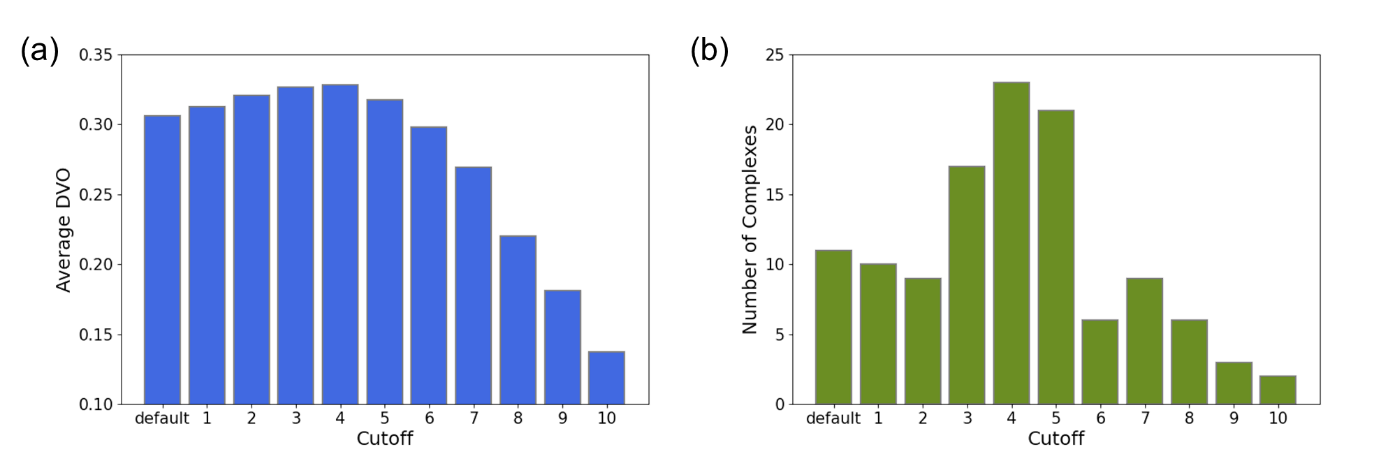


Figure S1. Analysis of alpha-spheres elimination at each cutoff. (a) Average Discretized Volume Overlap (DVO) for each cutoff. Each bar shows the average DVO of 117 complexes under each alpha sphere elimination condition. The cutoff corresponds to the severity of the process. The higher the average DVO, the more the refined pocket shape matches the ligand shape. (b) The number of complexes with maximum DVO for each cutoff. Each bar represents the number of complexes that formed the best pocket over another cutoff-based elimination. Note that the summation of all bars is 117, which is the number of complexes in the training dataset.

Table S1. Protein-ligand complexes in the training dataset and Discretized Volume Overlap (DVO) for each cutoff

| PDBID | $\frac{G_{\mathrm{pocket}}}{G_{\mathrm{ligand}}}$ | DVO for each cutoff | | | | | | | | | | |
| --- | --- | --- | --- | --- | --- | --- | --- | --- | --- | --- | --- | --- |
|  |  | 0 | 1 | 2 | 3 | 4 | 5 | 6 | 7 | 8 | 9 | 10 |
| 1e66 | 4.396 | 0.220 | 0.232 | 0.239 | 0.249 | 0.254 | 0.289 | 0.315 | 0.341 | 0.344 | 0.402 | 0.406 |
| 1eby | 2.143 | 0.372 | 0.372 | 0.361 | 0.352 | 0.354 | 0.290 | 0.230 | 0.229 | 0.211 | 0.196 | 0.174 |
| 1g2k | 2.041 | 0.381 | 0.388 | 0.399 | 0.385 | 0.389 | 0.415 | 0.430 | 0.406 | 0.262 | 0.262 | 0.258 |
| 1h22 | 3.047 | 0.289 | 0.294 | 0.308 | 0.317 | 0.339 | 0.337 | 0.365 | 0.370 | 0.401 | 0.447 | 0.336 |
| 1h23 | 2.331 | 0.392 | 0.401 | 0.430 | 0.442 | 0.424 | 0.402 | 0.445 | 0.438 | 0.430 | 0.403 | 0.404 |
| 1lpg | 1.492 | 0.475 | 0.485 | 0.469 | 0.441 | 0.430 | 0.399 | 0.438 | 0.437 | 0.455 | 0.387 | 0.390 |
| 1mq6 | 1.917 | 0.417 | 0.439 | 0.427 | 0.451 | 0.452 | 0.449 | 0.410 | 0.421 | 0.410 | 0.377 | 0.322 |
| 1nvq | 1.929 | 0.389 | 0.397 | 0.416 | 0.414 | 0.421 | 0.418 | 0.439 | 0.455 | 0.387 | 0.316 | 0.316 |
| 1o3f | 1.581 | 0.358 | 0.330 | 0.330 | 0.327 | 0.329 | 0.347 | 0.341 | 0.378 | 0.280 | 0.247 | 0.187 |
| 1owh | 2.924 | 0.237 | 0.237 | 0.231 | 0.221 | 0.195 | 0.196 | 0.228 | 0.215 | 0.226 | 0.233 | 0.228 |
| 1oyt | 3.533 | 0.243 | 0.246 | 0.251 | 0.262 | 0.263 | 0.250 | 0.230 | 0.217 | 0.228 | 0.242 | 0.252 |
| 1pxn | 6.955 | 0.125 | 0.126 | 0.127 | 0.129 | 0.136 | 0.143 | 0.146 | 0.121 | 0.139 | 0.103 | 0.071 |
| 1qf1 | 2.599 | 0.303 | 0.310 | 0.316 | 0.328 | 0.349 | 0.327 | 0.320 | 0.246 | 0.184 | 0.166 | 0.151 |
| 1qkt | 2.256 | 0.396 | 0.397 | 0.410 | 0.437 | 0.456 | 0.325 | 0.325 | 0.389 | 0.279 | 0.279 | 0.280 |
| 1sqa | 1.867 | 0.347 | 0.334 | 0.349 | 0.371 | 0.372 | 0.419 | 0.373 | 0.323 | 0.306 | 0.304 | 0.304 |
| 1u1b | 1.724 | 0.326 | 0.311 | 0.301 | 0.305 | 0.297 | 0.270 | 0.206 | 0.139 | 0.132 | 0.132 | 0.053 |
| 1y6r | 1.830 | 0.474 | 0.474 | 0.486 | 0.486 | 0.546 | 0.547 | 0.554 | 0.492 | 0.492 | 0.514 | 0.514 |
| 1ydt | 3.651 | 0.231 | 0.236 | 0.236 | 0.249 | 0.259 | 0.260 | 0.253 | 0.247 | 0.238 | 0.277 | 0.174 |
| 1z6e | 2.184 | 0.339 | 0.346 | 0.358 | 0.359 | 0.383 | 0.346 | 0.323 | 0.304 | 0.328 | 0.353 | 0.338 |
| 1z95 | 2.119 | 0.434 | 0.434 | 0.426 | 0.426 | 0.432 | 0.479 | 0.477 | 0.477 | 0.520 | 0.489 | 0.284 |
| 2al5 | 1.745 | 0.443 | 0.443 | 0.464 | 0.464 | 0.464 | 0.464 | 0.474 | 0.478 | 0.478 | 0.503 | 0.514 |
| 2c3i | 2.578 | 0.355 | 0.355 | 0.353 | 0.372 | 0.372 | 0.430 | 0.385 | 0.310 | 0.349 | 0.267 | 0.174 |
| 2cet | 2.902 | 0.284 | 0.284 | 0.284 | 0.305 | 0.315 | 0.317 | 0.279 | 0.312 | 0.291 | 0.291 | 0.122 |
| 2fvd | 4.828 | 0.174 | 0.175 | 0.176 | 0.178 | 0.173 | 0.140 | 0.179 | 0.214 | 0.211 | 0.179 | 0.206 |
| 2j7h | 6.155 | 0.128 | 0.128 | 0.128 | 0.136 | 0.137 | 0.147 | 0.189 | 0.315 | 0.288 | 0.290 | 0.000 |
| 2p15 | 1.745 | 0.524 | 0.529 | 0.529 | 0.559 | 0.581 | 0.615 | 0.595 | 0.566 | 0.490 | 0.479 | 0.421 |
| 2p4y | 2.953 | 0.298 | 0.302 | 0.303 | 0.298 | 0.297 | 0.315 | 0.334 | 0.310 | 0.317 | 0.174 | 0.091 |
| 2pog | 1.948 | 0.447 | 0.447 | 0.463 | 0.476 | 0.515 | 0.541 | 0.612 | 0.576 | 0.549 | 0.547 | 0.497 |
| 2qbp | 2.076 | 0.422 | 0.428 | 0.418 | 0.433 | 0.425 | 0.421 | 0.369 | 0.347 | 0.365 | 0.329 | 0.224 |
| 2qbq | 2.216 | 0.372 | 0.387 | 0.427 | 0.407 | 0.379 | 0.312 | 0.304 | 0.278 | 0.151 | 0.151 | 0.152 |
| 2qe4 | 1.913 | 0.470 | 0.477 | 0.502 | 0.502 | 0.539 | 0.573 | 0.528 | 0.535 | 0.517 | 0.463 | 0.084 |
| 2v7a | 2.947 | 0.271 | 0.270 | 0.278 | 0.284 | 0.279 | 0.301 | 0.258 | 0.218 | 0.241 | 0.102 | 0.107 |
| 2vkm | 3.442 | 0.254 | 0.254 | 0.264 | 0.275 | 0.283 | 0.292 | 0.345 | 0.340 | 0.364 | 0.419 | 0.442 |
| 2vvn | 3.096 | 0.274 | 0.274 | 0.284 | 0.292 | 0.305 | 0.346 | 0.368 | 0.374 | 0.494 | 0.498 | 0.462 |
| 2vw5 | 2.184 | 0.325 | 0.328 | 0.336 | 0.341 | 0.341 | 0.368 | 0.333 | 0.256 | 0.211 | 0.116 | 0.130 |
| 2wer | 3.006 | 0.269 | 0.280 | 0.290 | 0.306 | 0.318 | 0.344 | 0.321 | 0.316 | 0.330 | 0.285 | 0.000 |
| 2wn9 | 6.138 | 0.141 | 0.144 | 0.149 | 0.154 | 0.158 | 0.165 | 0.177 | 0.154 | 0.154 | 0.133 | 0.152 |
| 2wtv | 4.559 | 0.187 | 0.190 | 0.196 | 0.197 | 0.213 | 0.200 | 0.164 | 0.170 | 0.199 | 0.186 | 0.179 |
| 2x00 | 2.661 | 0.326 | 0.327 | 0.338 | 0.345 | 0.346 | 0.337 | 0.296 | 0.306 | 0.257 | 0.226 | 0.184 |
| 2xb8 | 2.552 | 0.339 | 0.339 | 0.362 | 0.355 | 0.369 | 0.382 | 0.357 | 0.400 | 0.410 | 0.423 | 0.433 |
| 2xbv | 1.980 | 0.392 | 0.392 | 0.404 | 0.407 | 0.436 | 0.446 | 0.456 | 0.451 | 0.487 | 0.380 | 0.370 |
| 2xii | 1.974 | 0.357 | 0.357 | 0.352 | 0.339 | 0.325 | 0.354 | 0.393 | 0.393 | 0.281 | 0.259 | 0.179 |
| 2xys | 4.686 | 0.197 | 0.199 | 0.207 | 0.215 | 0.227 | 0.226 | 0.232 | 0.257 | 0.276 | 0.313 | 0.354 |
| 2yki | 2.962 | 0.310 | 0.305 | 0.317 | 0.326 | 0.333 | 0.372 | 0.387 | 0.408 | 0.405 | 0.304 | 0.289 |
| 2zcq | 4.912 | 0.166 | 0.168 | 0.171 | 0.174 | 0.187 | 0.175 | 0.168 | 0.182 | 0.186 | 0.184 | 0.180 |
| 2zda | 3.099 | 0.250 | 0.257 | 0.265 | 0.267 | 0.266 | 0.254 | 0.272 | 0.236 | 0.245 | 0.197 | 0.198 |
| 2zy1 | 4.789 | 0.185 | 0.189 | 0.190 | 0.193 | 0.197 | 0.206 | 0.208 | 0.252 | 0.309 | 0.321 | 0.235 |
| 3ag9 | 2.175 | 0.220 | 0.228 | 0.235 | 0.226 | 0.222 | 0.215 | 0.211 | 0.221 | 0.194 | 0.153 | 0.151 |
| 3arp | 5.992 | 0.141 | 0.142 | 0.145 | 0.151 | 0.159 | 0.140 | 0.139 | 0.134 | 0.135 | 0.143 | 0.113 |
| 3b1m | 4.042 | 0.216 | 0.216 | 0.219 | 0.213 | 0.216 | 0.229 | 0.184 | 0.170 | 0.125 | 0.134 | 0.109 |
| 3b5r | 2.278 | 0.409 | 0.418 | 0.430 | 0.438 | 0.438 | 0.447 | 0.515 | 0.584 | 0.504 | 0.475 | 0.460 |
| 3b65 | 2.376 | 0.399 | 0.403 | 0.403 | 0.399 | 0.430 | 0.430 | 0.460 | 0.435 | 0.431 | 0.421 | 0.352 |
| 3b68 | 2.053 | 0.450 | 0.455 | 0.455 | 0.470 | 0.490 | 0.514 | 0.576 | 0.561 | 0.570 | 0.472 | 0.436 |
| 3dd0 | 2.646 | 0.347 | 0.361 | 0.398 | 0.394 | 0.371 | 0.366 | 0.367 | 0.315 | 0.287 | 0.141 | 0.169 |
| 3e5a | 2.126 | 0.373 | 0.373 | 0.385 | 0.437 | 0.398 | 0.433 | 0.383 | 0.302 | 0.302 | 0.284 | 0.240 |
| 3e92 | 2.948 | 0.309 | 0.309 | 0.318 | 0.318 | 0.336 | 0.400 | 0.429 | 0.431 | 0.460 | 0.488 | 0.297 |
| 3e93 | 3.783 | 0.227 | 0.227 | 0.228 | 0.240 | 0.238 | 0.252 | 0.230 | 0.196 | 0.211 | 0.223 | 0.263 |
| 3ejr | 1.208 | 0.410 | 0.430 | 0.430 | 0.421 | 0.476 | 0.357 | 0.353 | 0.328 | 0.328 | 0.328 | 0.302 |
| 3f3d | 1.752 | 0.406 | 0.406 | 0.406 | 0.406 | 0.406 | 0.428 | 0.428 | 0.447 | 0.464 | 0.385 | 0.389 |
| 3f3e | 2.062 | 0.379 | 0.379 | 0.379 | 0.379 | 0.379 | 0.396 | 0.442 | 0.442 | 0.442 | 0.331 | 0.315 |
| 3fur | 4.162 | 0.222 | 0.222 | 0.222 | 0.229 | 0.236 | 0.240 | 0.285 | 0.272 | 0.274 | 0.247 | 0.231 |
| 3fv1 | 3.660 | 0.223 | 0.223 | 0.223 | 0.223 | 0.225 | 0.247 | 0.265 | 0.262 | 0.324 | 0.301 | 0.445 |
| 3fv2 | 6.459 | 0.135 | 0.136 | 0.137 | 0.146 | 0.148 | 0.160 | 0.180 | 0.195 | 0.272 | 0.284 | 0.301 |
| 3g0w | 3.256 | 0.273 | 0.273 | 0.275 | 0.275 | 0.282 | 0.288 | 0.314 | 0.332 | 0.344 | 0.429 | 0.489 |
| 3gc5 | 2.236 | 0.389 | 0.389 | 0.410 | 0.426 | 0.440 | 0.525 | 0.545 | 0.546 | 0.514 | 0.399 | 0.319 |
| 3ge7 | 4.164 | 0.204 | 0.206 | 0.213 | 0.231 | 0.247 | 0.270 | 0.281 | 0.246 | 0.256 | 0.244 | 0.159 |
| 3gnw | 3.706 | 0.210 | 0.205 | 0.191 | 0.197 | 0.192 | 0.191 | 0.204 | 0.193 | 0.126 | 0.092 | 0.065 |
| 3kr8 | 4.392 | 0.209 | 0.209 | 0.223 | 0.238 | 0.247 | 0.265 | 0.286 | 0.281 | 0.315 | 0.258 | 0.276 |
| 3myg | 2.981 | 0.259 | 0.263 | 0.274 | 0.274 | 0.276 | 0.287 | 0.285 | 0.192 | 0.203 | 0.084 | 0.093 |
| 3nw9 | 1.555 | 0.444 | 0.458 | 0.458 | 0.455 | 0.470 | 0.449 | 0.461 | 0.377 | 0.323 | 0.212 | 0.218 |
| 3nx7 | 2.860 | 0.216 | 0.216 | 0.216 | 0.238 | 0.275 | 0.304 | 0.296 | 0.309 | 0.327 | 0.333 | 0.376 |
| 3o9i | 2.470 | 0.344 | 0.352 | 0.357 | 0.362 | 0.395 | 0.406 | 0.351 | 0.273 | 0.323 | 0.325 | 0.353 |
| 3oe4 | 1.980 | 0.414 | 0.414 | 0.419 | 0.430 | 0.438 | 0.463 | 0.419 | 0.400 | 0.362 | 0.395 | 0.257 |
| 3p5o | 3.049 | 0.259 | 0.257 | 0.266 | 0.252 | 0.234 | 0.200 | 0.114 | 0.069 | 0.048 | 0.000 | 0.000 |
| 3prs | 1.632 | 0.429 | 0.432 | 0.429 | 0.405 | 0.415 | 0.399 | 0.388 | 0.334 | 0.360 | 0.202 | 0.196 |
| 3pww | 2.194 | 0.326 | 0.342 | 0.355 | 0.365 | 0.381 | 0.343 | 0.329 | 0.365 | 0.263 | 0.154 | 0.114 |
| 3qgy | 5.664 | 0.144 | 0.147 | 0.149 | 0.142 | 0.118 | 0.093 | 0.080 | 0.078 | 0.098 | 0.039 | 0.000 |
| 3rlr | 2.613 | 0.349 | 0.349 | 0.365 | 0.373 | 0.378 | 0.405 | 0.328 | 0.306 | 0.210 | 0.203 | 0.000 |
| 3ryj | 2.227 | 0.317 | 0.330 | 0.334 | 0.343 | 0.335 | 0.270 | 0.236 | 0.201 | 0.145 | 0.136 | 0.136 |
| 3tsk | 2.057 | 0.359 | 0.366 | 0.368 | 0.375 | 0.389 | 0.399 | 0.388 | 0.347 | 0.330 | 0.324 | 0.332 |
| 3u8k | 3.685 | 0.244 | 0.251 | 0.251 | 0.251 | 0.252 | 0.304 | 0.340 | 0.405 | 0.411 | 0.391 | 0.391 |
| 3u8n | 4.631 | 0.203 | 0.203 | 0.208 | 0.210 | 0.216 | 0.254 | 0.271 | 0.297 | 0.357 | 0.351 | 0.192 |
| 3ui7 | 4.104 | 0.199 | 0.204 | 0.202 | 0.204 | 0.211 | 0.218 | 0.191 | 0.140 | 0.144 | 0.195 | 0.108 |
| 3up2 | 2.275 | 0.372 | 0.372 | 0.378 | 0.386 | 0.377 | 0.381 | 0.291 | 0.247 | 0.250 | 0.270 | 0.278 |
| 3uri | 1.481 | 0.346 | 0.340 | 0.332 | 0.326 | 0.334 | 0.339 | 0.347 | 0.274 | 0.180 | 0.156 | 0.154 |
| 3utu | 2.012 | 0.340 | 0.346 | 0.331 | 0.320 | 0.312 | 0.310 | 0.298 | 0.277 | 0.282 | 0.293 | 0.303 |
| 3uuo | 3.362 | 0.225 | 0.225 | 0.232 | 0.236 | 0.234 | 0.245 | 0.240 | 0.256 | 0.177 | 0.122 | 0.119 |
| 3zdg | 4.005 | 0.233 | 0.233 | 0.233 | 0.238 | 0.269 | 0.275 | 0.288 | 0.400 | 0.412 | 0.282 | 0.189 |
| 4cra | 1.525 | 0.461 | 0.480 | 0.440 | 0.417 | 0.396 | 0.379 | 0.323 | 0.255 | 0.236 | 0.236 | 0.236 |
| 4crc | 2.360 | 0.336 | 0.344 | 0.360 | 0.365 | 0.323 | 0.328 | 0.302 | 0.300 | 0.307 | 0.247 | 0.254 |
| 4e5w | 4.353 | 0.208 | 0.216 | 0.216 | 0.229 | 0.241 | 0.230 | 0.231 | 0.238 | 0.205 | 0.164 | 0.000 |
| 4e6q | 2.113 | 0.435 | 0.435 | 0.435 | 0.436 | 0.442 | 0.449 | 0.456 | 0.443 | 0.387 | 0.266 | 0.135 |
| 4eo8 | 1.451 | 0.473 | 0.496 | 0.496 | 0.492 | 0.470 | 0.469 | 0.400 | 0.408 | 0.368 | 0.345 | 0.232 |
| 4f2w | 2.087 | 0.429 | 0.429 | 0.430 | 0.437 | 0.463 | 0.480 | 0.496 | 0.496 | 0.468 | 0.458 | 0.511 |
| 4f3c | 1.886 | 0.466 | 0.466 | 0.485 | 0.495 | 0.551 | 0.572 | 0.572 | 0.595 | 0.602 | 0.593 | 0.602 |
| 4gfm | 2.858 | 0.323 | 0.323 | 0.332 | 0.354 | 0.365 | 0.392 | 0.430 | 0.442 | 0.373 | 0.321 | 0.373 |
| 4gid | 2.716 | 0.332 | 0.340 | 0.365 | 0.376 | 0.394 | 0.403 | 0.398 | 0.391 | 0.390 | 0.399 | 0.318 |
| 4gr0 | 2.124 | 0.362 | 0.362 | 0.353 | 0.365 | 0.395 | 0.395 | 0.398 | 0.368 | 0.337 | 0.253 | 0.212 |
| 4hge | 2.795 | 0.323 | 0.323 | 0.341 | 0.351 | 0.358 | 0.374 | 0.341 | 0.377 | 0.330 | 0.326 | 0.172 |
| 4ivb | 4.533 | 0.196 | 0.200 | 0.209 | 0.216 | 0.221 | 0.233 | 0.276 | 0.153 | 0.137 | 0.157 | 0.157 |
| 4ivc | 6.736 | 0.140 | 0.139 | 0.142 | 0.148 | 0.150 | 0.163 | 0.204 | 0.196 | 0.224 | 0.239 | 0.200 |
| 4ivd | 3.121 | 0.295 | 0.302 | 0.316 | 0.330 | 0.347 | 0.379 | 0.479 | 0.415 | 0.294 | 0.300 | 0.295 |
| 4j21 | 3.246 | 0.276 | 0.262 | 0.262 | 0.260 | 0.288 | 0.316 | 0.320 | 0.328 | 0.366 | 0.395 | 0.213 |
| 4j3l | 4.005 | 0.197 | 0.199 | 0.194 | 0.194 | 0.199 | 0.205 | 0.192 | 0.210 | 0.218 | 0.206 | 0.225 |
| 4jia | 5.941 | 0.133 | 0.133 | 0.128 | 0.132 | 0.141 | 0.114 | 0.111 | 0.109 | 0.087 | 0.059 | 0.068 |
| 4k18 | 2.781 | 0.321 | 0.324 | 0.343 | 0.365 | 0.369 | 0.362 | 0.411 | 0.378 | 0.407 | 0.213 | 0.123 |
| 4pcs | 1.651 | 0.323 | 0.305 | 0.305 | 0.316 | 0.298 | 0.285 | 0.262 | 0.262 | 0.251 | 0.251 | 0.287 |
| 4qac | 7.210 | 0.129 | 0.130 | 0.135 | 0.139 | 0.153 | 0.166 | 0.190 | 0.231 | 0.281 | 0.424 | 0.454 |
| 4qd6 | 2.550 | 0.214 | 0.183 | 0.164 | 0.147 | 0.147 | 0.122 | 0.122 | 0.075 | 0.085 | 0.095 | 0.095 |
| 4rfm | 2.850 | 0.269 | 0.270 | 0.262 | 0.221 | 0.260 | 0.261 | 0.295 | 0.291 | 0.287 | 0.221 | 0.000 |
| 4tmn | 2.443 | 0.340 | 0.355 | 0.355 | 0.373 | 0.385 | 0.412 | 0.344 | 0.354 | 0.355 | 0.237 | 0.234 |
| 4twp | 2.729 | 0.302 | 0.308 | 0.361 | 0.385 | 0.392 | 0.424 | 0.421 | 0.450 | 0.421 | 0.442 | 0.350 |
| 4ty7 | 2.163 | 0.329 | 0.339 | 0.339 | 0.353 | 0.320 | 0.273 | 0.215 | 0.161 | 0.162 | 0.176 | 0.191 |
| 4x6p | 2.472 | 0.317 | 0.326 | 0.309 | 0.314 | 0.298 | 0.283 | 0.214 | 0.229 | 0.231 | 0.246 | 0.250 |
| 5c2h | 3.467 | 0.260 | 0.260 | 0.263 | 0.291 | 0.293 | 0.279 | 0.285 | 0.286 | 0.249 | 0.229 | 0.279 |
| 5dwr | 1.850 | 0.435 | 0.435 | 0.468 | 0.468 | 0.477 | 0.490 | 0.515 | 0.446 | 0.396 | 0.325 | 0.329 |
| 5tmn | 5.888 | 0.127 | 0.124 | 0.126 | 0.133 | 0.146 | 0.145 | 0.145 | 0.139 | 0.103 | 0.113 | 0.073 |
| **Average** | **3.032** | **0.306** | **0.313** | **0.321** | **0.327** | **0.328** | **0.317** | **0.298** | **0.270** | **0.220** | **0.181** | **0.137** |

**Appendix 3: Performance**

The validity of the optimized process was evaluated using the test dataset, which included 83 protein-ligand complexes (Table S2) from the Astex dataset that did not overlap with the training dataset (Hartshorn *et al.*, 2007). The test dataset was prepared in the same way as the training dataset. The P2C process with optimized parameters was applied to the test dataset. The DVO was calculated before and after the alpha-spheres elimination process in the same way as the training dataset. Additionally, the differences in the DVO before and after the process was calculated for each complex and termed $\Delta$DVO. Figure S2 shows the DVO improvement distribution before and after the elimination process; an improved DVO after the process implies a positive $\Delta$DVO value (Table S2). Approximately 80% of complexes (66/83) in the test dataset showed an improvement in DVO. For most of the complexes, it indicates that alpha-spheres elimination works to fit the shape of the pocket and ligand. This result suggests that the P2C process is effective for the training dataset and diverse complexes.


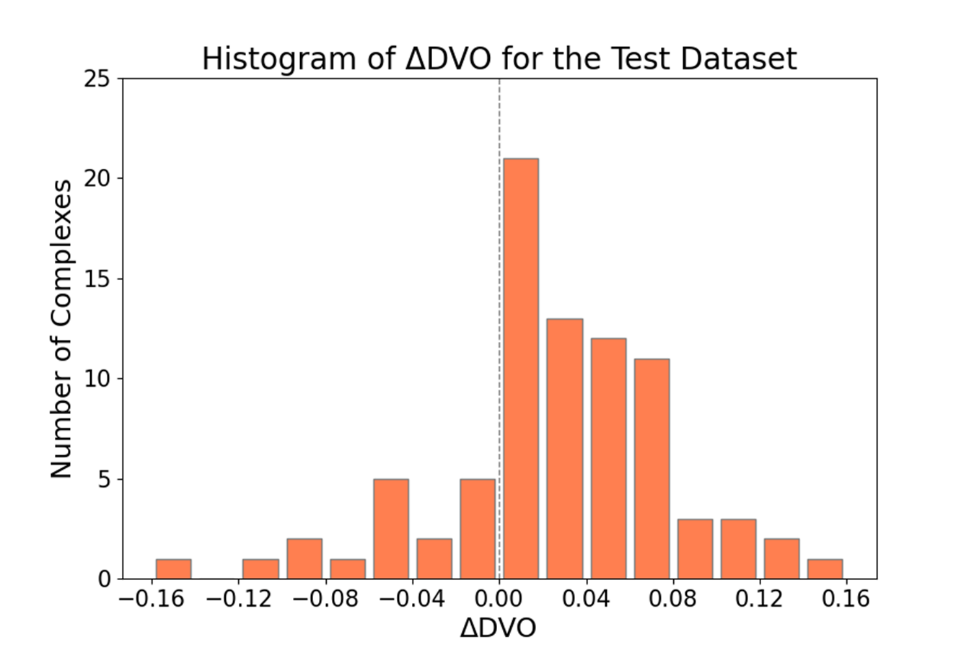


Figure S2. Histograms showing the difference in Discretized Volume Overlap ($\Delta$DVO) before and after the elimination of alpha-spheres for the test dataset. The width per bar is 0.02, and a dotted line separates the positive and negative values of ∆DVO.

Figure S3 shows three complexes of the test dataset and alpha spheres with improved DVO. The complex of thiamin pyrophosphokinase and thiamin (Timm *et al.*, 2001) showed the best $\Delta$DVO in the test dataset (before: 0.263; after: 0.412; Figure S3a). P2C removed alpha spheres far from the ligand, and those at the ligand position were well conserved. Figure S3b depicts the complex structure of alpha-mannosidase and swainsonine (Timm *et al.*, 2001) with the second-best $\Delta$DVO in the test dataset (before: 0.495; after: 0.631; Figure S3b). Alpha spheres that extended away from the ligand were deleted, and those at the ligand position were precisely conserved. This result suggests that the P2C process could accurately form the pocket at the binding site, even for a ligand comprising a small fragment of swainsonine. The complex of prostaglandin synthase and its ligand (Gupta *et al.*, 2004) showed the third-best $\Delta$DVO in the test dataset (before: 0.205; after: 0.327; Figure S3c). For this complex, the P2C process modified the overall shape of the alpha spheres around the ligand into a fitted pocket, preserving only those closest to the ligand position.


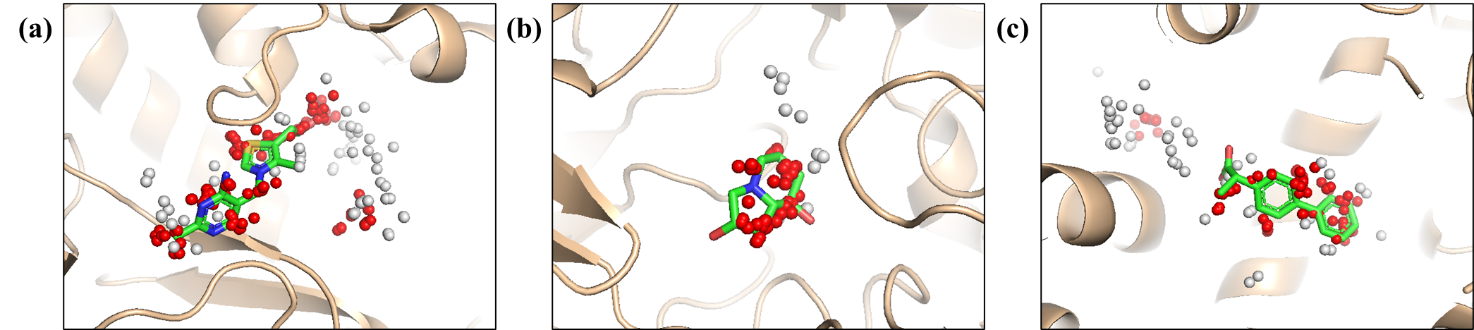


Figure S3. Structural representation of the complexes in the test dataset with the highest $\Delta$DVO. (a) Complex (PDB ID: 1IG3) with the highest $\Delta$DVO. (b) Complex (PDB ID: 1HWW) with the second-highest $\Delta$DVO. (c) Complex (PDB ID: 1Q4G) with the third-highest $\Delta$DVO. Proteins, ligands, and deleted and remaining alpha spheres are shown as a cartoon, stick, and white and red balls, respectively.

Structures of some complexes with a negative $\Delta$DVO value are illustrated in Figure S4. In the complex of ADAM33 and marimastat (Orth *et al.*, 2004) with the lowest $\Delta$DVO in the test dataset (before: 0.353; after: 0.197; Figure S4a), alpha spheres entirely overlapped the ligand before the process, whereas alpha spheres were greatly reduced after the process, and overlapped only a part of the ligand (Figure S4a). Figure S4b shows the complex structure of DPP4 and its inhibitor (Rasmussen *et al.*, 2003) with the second-lowest $\Delta$DVO in the test dataset (before: 0.383; after: 0.282; Figure S4b). The pyrrolidine moiety in the ligand overlapped with the remaining alpha spheres, whereas alpha spheres near other parts of the ligand were removed due to alpha-spheres elimination. The complex of penicillin acylase and oxidized penicillin G (McVey *et al.*, 2001) showed the third-lowest $\Delta$DVO in the test dataset (before: 0.252; after: 0.160; Figure S4c). The exclusive benzyl moiety in the ligand did not overlap with the alpha spheres after the process.

Additionally, the ligand interaction diagrams using MOE (Chemical Computing Group, LLC) showed that alpha spheres located on the chemical structure in the solvent exposure area were often eliminated after the process (Figure S4a–c). The solvent exposure ratio of the ligand was calculated for the test dataset to analyze the relationship between the solvent exposure of the ligand and the effect of the alpha sphere elimination. The scatter plot between the solvent exposure ratio of the ligand and ΔDVO is presented in Figure S5, which indicates that the complex with a high solvent exposure ligand ratio has low ΔDVO. The average exposure ratio of complexes with negative ΔDVO was 0.128, and that of complexes with positive ΔDVO was 0.025. Therefore, the deeper the ligand is in the protein pocket, the more effective the P2C process is.

On the contrary, the more the ligand is exposed to the solvent, the less effective the process becomes. This result is due to the conditions of alpha-spheres formation in the generator. Alpha-spheres are generated by concentrating on the concavity of higher curvature in the protein. A few alpha-spheres are located on the protein surface, largely exposed to the solvent. This result suggests that alpha spheres elimination does not follow the shape of the ligand on the surface. However, the shape of the ligand on the concavity of the protein is followed by the alpha-spheres elimination process.


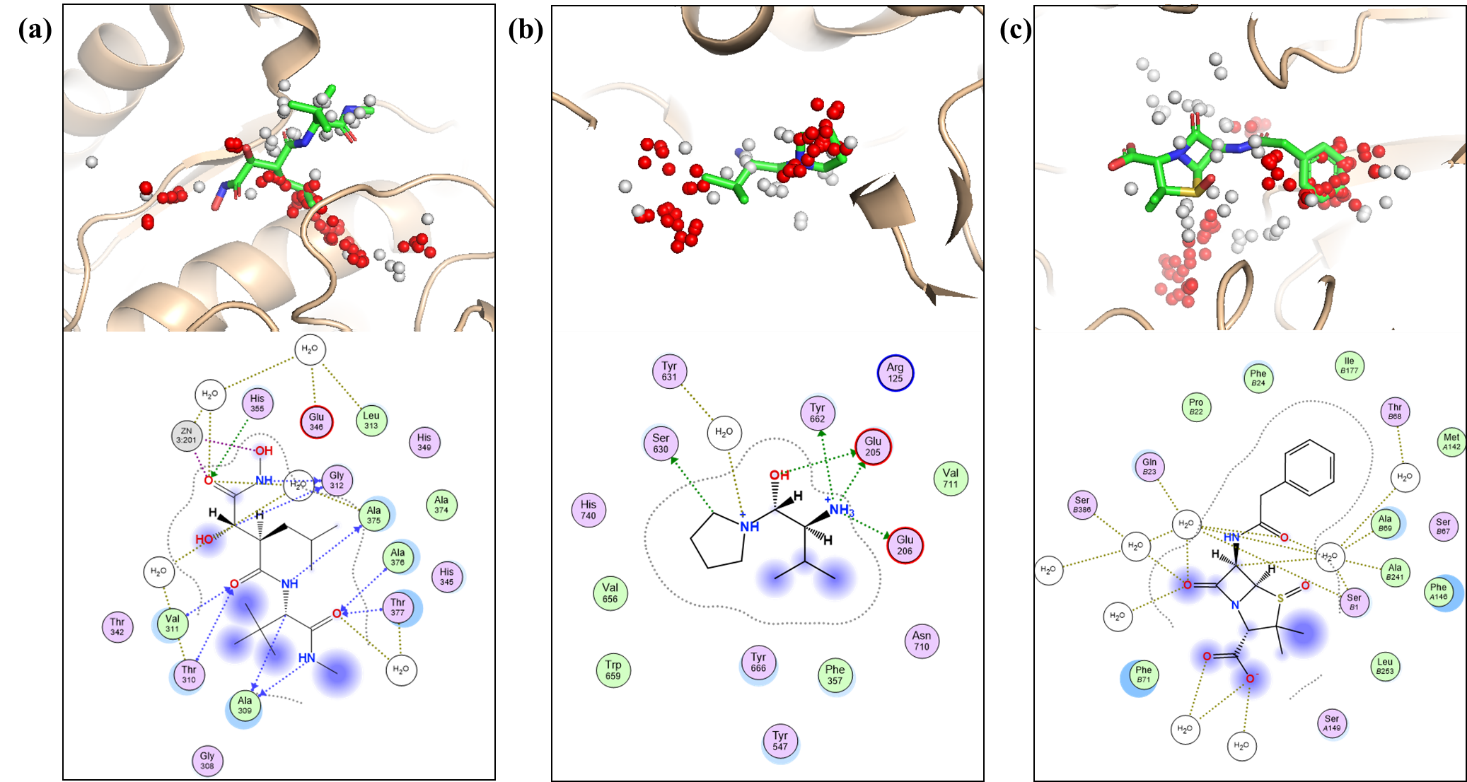


Figure S4. Structural representation of the complexes with the lowest ΔDVO in the test dataset. (a) Complex (PDB ID: 1R55) with the lowest ΔDVO. (b) Complex (PDB ID: 1N1M) with the second-lowest ΔDVO. (c) Complex (PDB ID: 1GM8) with the third-lowest ΔDVO. The bottom part of each figure shows the 2D diagram of the ligand exposure. Ligands covered with an indigo veil denote the solvent-accessible area.


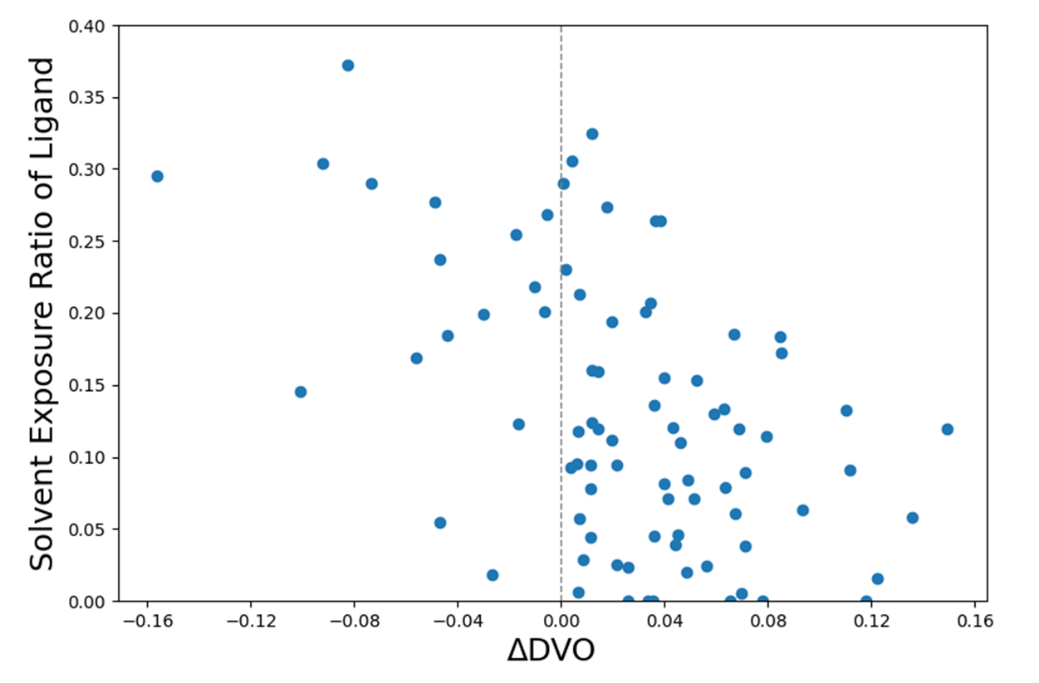


Figure S5. Scatter plot between the solvent exposure ratio and ΔDVO for the test dataset. A dotted line separates the positive and negative ∆DVO values.

Table S2. Complexes in the test dataset and Discretized Volume Overlap (DVO) before and after alpha-spheres elimination

| PDBID | Protein | Ligand | Used Chain ID | DVO  (before) | DVO  (after) | ΔDVO |
| --- | --- | --- | --- | --- | --- | --- |
| 1g9v | deoxy hemoglobin | RQ3 | A | 0.095 | 0.096 | 0.001 |
| 1gkc | matrix metalloprotease 9 | NFH | A | 0.257 | 0.208 | -0.049 |
| 1gm8 | penicillin G acylase | SOX | B | 0.252 | 0.160 | -0.092 |
| 1gpk | acetylcholinesterase | HUP | A | 0.449 | 0.516 | 0.067 |
| 1hnn | phenyl ethanolamine N-methyltransferase | SKF | A | 0.152 | 0.178 | 0.026 |
| 1hp0 | purine specific nucleoside hydrolase | AD3 | A | 0.473 | 0.529 | 0.056 |
| 1hq2 | 6-hydroxymethyl-7, 8-dihydropterin pyrophosphokinase | PH2 | A | 0.163 | 0.212 | 0.049 |
| 1hvy | thymidylate synthase | D16 | A | 0.140 | 0.142 | 0.002 |
| 1hwi | HMG-CoA reductase | 115 | A | 0.345 | 0.289 | -0.056 |
| 1hww | α-mannosidase Ⅱ | SWA | A | 0.495 | 0.631 | 0.136 |
| 1ia1 | dihydrofolate reductase | TQ3 | A | 0.122 | 0.134 | 0.012 |
| 1ig3 | thiamin pyrophosphokinase | VIB | A | 0.263 | 0.412 | 0.149 |
| 1j3j | dihydrofolate reductase | CP6 | A | 0.121 | 0.135 | 0.014 |
| 1jd0 | carbonic anhydrase Ⅻ | AZM | A | 0.258 | 0.343 | 0.085 |
| 1jje | metallo β-lactamase | BYS | A | 0.366 | 0.374 | 0.007 |
| 1jla | HIV-1 reverse transcriptase | TNK | A | 0.125 | 0.147 | 0.022 |
| 1k3u | tryptophan synthase | IAD | A | 0.418 | 0.536 | 0.118 |
| 1ke5 | cyclin-dependent kinase 2 | LS1 | A | 0.183 | 0.235 | 0.053 |
| 1kzk | HIV-1 protease | JE2 | A | 0.353 | 0.424 | 0.071 |
| 1l2s | β-lactamase | STC | A | 0.109 | 0.092 | -0.018 |
| 1l7f | neuraminidase A | BCZ | A | 0.432 | 0.416 | -0.016 |
| 1lpz | factor Ⅹa | CMB | B | 0.376 | 0.366 | -0.010 |
| 1lrh | auxin-binding protein 1 | NLA | A | 0.242 | 0.291 | 0.048 |
| 1m2z | glucocorticoid receptor | DEX | A | 0.506 | 0.513 | 0.007 |
| 1meh | inosine monophosphate dehydrogenase | MOA | A | 0.111 | 0.123 | 0.012 |
| 1mmv | neuronal nitric-oxide synthase | 3AR | A | 0.109 | 0.148 | 0.039 |
| 1mzc | protein farnesyltransferase | BNE | B | 0.218 | 0.136 | -0.083 |
| 1n1m | dipeptidyl peptidase Ⅳ | A3M | A | 0.383 | 0.282 | -0.101 |
| 1n2j | pantothenate synthetase | PAF | A | 0.065 | 0.085 | 0.020 |
| 1n2v | tRNA-guanine transglycosylase | BDI | A | 0.279 | 0.346 | 0.067 |
| 1n46 | thyroid hormone receptor β1 | PFA | A | 0.433 | 0.469 | 0.036 |
| 1nav | thyroid hormone receptor α1 | IH5 | A | 0.329 | 0.303 | -0.027 |
| 1of1 | thymidine kinase | SCT | A | 0.214 | 0.279 | 0.065 |
| 1of6 | DAHP synthase | DTY | A | 0.484 | 0.562 | 0.078 |
| 1opk | c-Ab1 tyrosine kinase | P16 | A | 0.414 | 0.494 | 0.080 |
| 1oq5 | carbonic anhydrase Ⅱ | CEL | A | 0.289 | 0.282 | -0.006 |
| 1owe | urokinase | 675 | A | 0.220 | 0.224 | 0.004 |
| 1p2y | cytochrome P450cam | NCT | A | 0.267 | 0.302 | 0.035 |
| 1p62 | deoxycytidine kinase | GEO | B | 0.469 | 0.514 | 0.045 |
| 1pmn | c-Jun terminal kinase 3 | 984 | A | 0.241 | 0.274 | 0.033 |
| 1q1g | purine nucleoside phosphorylase | MTI | A | 0.191 | 0.227 | 0.036 |
| 1q41 | glycogen synthase kinase 3β | IXM | A | 0.337 | 0.449 | 0.112 |
| 1q4g | prostaglandin H2 synthase 1 | BFL | A | 0.205 | 0.327 | 0.122 |
| 1r1h | neprilysin | BIR | A | 0.319 | 0.362 | 0.043 |
| 1r55 | ADAM33 | 97 | A | 0.353 | 0.197 | -0.156 |
| 1r58 | methionine aminopeptidase 2 | AO5 | A | 0.319 | 0.272 | -0.047 |
| 1r9o | cytochrome P450 2C9 | FLP | A | 0.131 | 0.143 | 0.012 |
| 1s19 | vitamin D nuclear receptor | MC9 | A | 0.402 | 0.472 | 0.070 |
| 1s3v | dihydrofolate reductase | TQD | A | 0.261 | 0.297 | 0.036 |
| 1sg0 | quinone reductase 2 | STL | A | 0.243 | 0.261 | 0.018 |
| 1sj0 | estrogen receptor α | E4D | A | 0.292 | 0.344 | 0.052 |
| 1sq5 | pantothenate kinase | PAU | A | 0.080 | 0.101 | 0.021 |
| 1sqn | progesterone receptor | NDR | A | 0.459 | 0.492 | 0.033 |
| 1t40 | aldose reductase | ID5 | A | 0.330 | 0.333 | 0.004 |
| 1t46 | c-kit tyrosine kinase | STI | A | 0.320 | 0.384 | 0.064 |
| 1t9b | acetohydroxyacid synthase | 1CS | A | 0.289 | 0.375 | 0.085 |
| 1tow | adipocyte fatty acid-binding protein | CRZ | A | 0.326 | 0.398 | 0.071 |
| 1tt1 | glutamate receptor 6 | KAI | A | 0.204 | 0.248 | 0.044 |
| 1tz8 | transthyretin | DES | B | 0.257 | 0.210 | -0.047 |
| 1u1c | uridine phosphorylase | BAU | A | 0.360 | 0.367 | 0.007 |
| 1u4d | activated Cdc42 kinase 1 | DBQ | A | 0.127 | 0.133 | 0.006 |
| 1uml | adenosine deaminase | FR4 | A | 0.309 | 0.321 | 0.011 |
| 1unl | cyclin-dependent kinase 5 | RRC | A | 0.244 | 0.304 | 0.059 |
| 1uou | thymidine phosphorylase | CMU | A | 0.313 | 0.322 | 0.009 |
| 1v0p | protein kinase 5 | PVB | A | 0.282 | 0.237 | -0.044 |
| 1v48 | purine nucleoside phosphorylase | HA1 | A | 0.409 | 0.450 | 0.041 |
| 1v4s | glucokinase | MRK | A | 0.286 | 0.380 | 0.093 |
| 1vcj | neuraminidase B | IBA | A | 0.366 | 0.412 | 0.046 |
| 1w1p | chitinase B | GIO | A | 0.045 | 0.051 | 0.006 |
| 1w2g | thymidylate kinase | THM | A | 0.352 | 0.392 | 0.040 |
| 1x8x | tyrosyl-tRNA synthetase | TYR | A | 0.171 | 0.240 | 0.069 |
| 1xm6 | phosphodiesterase 4B | 5RM | A | 0.221 | 0.232 | 0.011 |
| 1xoq | phosphodiesterase 4D | ROF | A | 0.220 | 0.283 | 0.063 |
| 1xoz | phosphodiesterase 5A | CIA | A | 0.246 | 0.260 | 0.014 |
| 1y6b | vascular endothelial growth factor receptor 2 | AAX | A | 0.485 | 0.504 | 0.019 |
| 1ygc | factor Ⅶa | 905 | H | 0.335 | 0.330 | -0.005 |
| 1yqy | lethal factor | 915 | A | 0.094 | 0.106 | 0.011 |
| 1yv3 | myosin Ⅱ | BIT | A | 0.076 | 0.101 | 0.026 |
| 1yvf | NS5B polymerase | PH7 | A | 0.171 | 0.141 | -0.030 |
| 1ywr | p38 kinase | LI9 | A | 0.256 | 0.296 | 0.040 |
| 2bm2 | βⅡtryptase | PM2 | A | 0.223 | 0.260 | 0.037 |
| 2br1 | Chk1 | PFP | A | 0.339 | 0.266 | -0.074 |
| 2bsm | heat shock protein 90 | BSM | A | 0.358 | 0.468 | 0.110 |

**Appendix 4: Application for Hit-to-Lead complexes**

P2C was applied to the pairs of complexes with hit and lead compounds, and the selected two complexes: BCL-XL and Thrombi, were downloaded from the PDB site (Orita *et al.*, 2009). LB mode in P2C was performed for complexes with hit compounds. The distance parameter in LB mode was set within the range, including the pocket that binds to the lead compound in this benchmark. Note that empty sites identification is not performed in the benchmark. The structure of the BCL-XL complex with hit and lead compounds is shown in Figure S6a. Hit and lead complex structures were aligned with the protein backbone. The lead compound was generated based on the hit compound using a structure-activity relationship (SAR) by nuclear magnetic resonance (NMR; Oltersdorf *et al.*, 2005). The structure of the thrombin complex with the hit compound is depicted in Figure S6b. Tetrazole and chlorobenzene moieties are common as hit and lead compounds. The lead compound is generated by fragment-linking between the hit compound and another hit fragment, which is not contained in the hit complex (Howard *et al.*, 2006). In both cases, alpha spheres were generated based on the complex, with the hit compound positioned as the hit compound and the lead compound. These results suggest that alpha-spheres after the P2C process also show an appropriate shape as the lead compound, and the P2C is available as the lead optimization guideline from the hit complex.


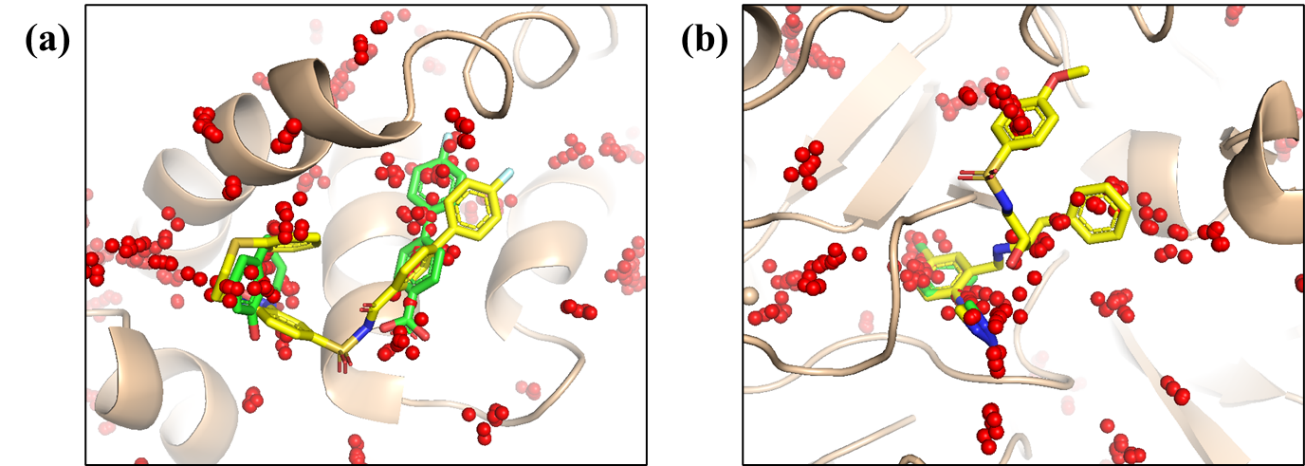


Figure S6. Application of Pocket to Concavity (P2C) for Hit-to-Lead complexes. (a) The complex of BCL-XL with hit compound after alpha sphere elimination. Hit and lead complexes are referred to as 1YSG and 1YSI (PDB IDs). (b) The complex of thrombin with hit compound after alpha-spheres elimination. Hit and lead complexes are referred to as 2C90 and 2C8W (PDB IDs). BCL-XL, the hit compound, and alpha-spheres after P2C are shown in the cartoon, green-colored stick, and red ball models, respectively. The yellow-colored stick model corresponds to the lead compound. After the process, the cartoon model of the protein and the ball model of the alpha-spheres was derived from the hit complex. The lead complex was aligned with the protein backbone, and the protein structure of the lead complex was deleted from the figure.

**Appendix 5: Case study**

β-ketoacyl-acyl carrier protein synthase Ш and the inhibitor (PDBID: 4Z8D) are used for a case study (McKinney *et al.*, 2016). Here there are four use cases; (A) LF mode (default), (B) LF mode (without alpha-spheres generation), (C) LB mode (default), and (D) LB mode (without alpha-spheres generation).

**(A). LF mode (default)**

The default LF mode provides the shape of the deep and druggable concavity where the core scaffold can bind. The command to run in this case is as follows:

$ p2c -m LF -p 4Z8D_protein.pdb -r 1

“4Z8D_protein.pdb” is the input protein structure file. P2C is available in a PDB format as the protein structure file. The optional argument “-r 1” specifies the number of pockets (sorted druggability score) that execute P2C processes.

**(B). LF mode (without alpha-spheres generation)**

LF mode can be performed to refine alpha-spheres from other software (e.g., output by SiteFiner). The command to run LF mode, in this case, is as follows:

$ p2c -m LF　-p 4Z8D_protein.pdb -a pockets.pqr -n 4

“pockets.pqr” is the alpha spheres coordinate file from other settings or alpha sphere-based software. Note that the re-optimized parameter should be used if another setting is used as the pockets file. The re-optimized parameter can be specified in the optional argument “-n”.

**(C). LB mode (default)**

Deep concavity around active ligands can be searched with LB mode in P2C. The command to run LB mode with alpha-sphere generation is as follows:

$ p2c -m LB -p 4Z8D_protein.pdb -l 4Z8D_ligand.pdb -d 10

“4Z8D_ligand.pdb” is the input protein structure file (PDB format). LB mode identified that the predicted pocket is occupied/unoccupied by a ligand using the coordination of the protein and ligand. The range of search is specified by “-d”. “-d 10” means LB mode searches unoccupied pockets within 10 Å from the ligand.

**(D). LB mode (without alpha-spheres generation)**

The LB mode can be performed to refine alpha-spheres from other software, the same as the LF mode. The command to run LB mode, in this case, is as follows:

$ p2c -m LB -p 4Z8D_protein.pdb -l 4Z8D_ligand.pdb -a pockets.pqr -n 4

“-a” is the same as LF mode without alpha-spheres generation. Note that a re-optimized parameter should be used if another default setting is used as the pocket file.


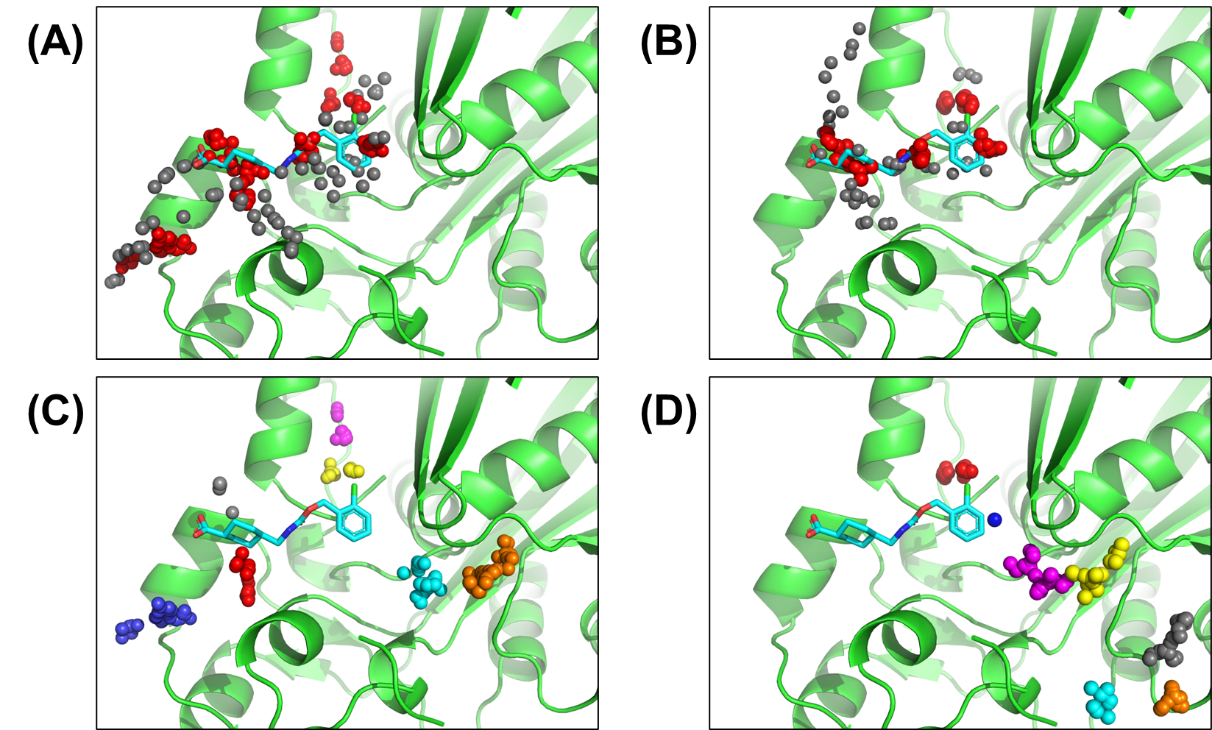


Figure S7. Use case for different modes and settings. (A) LF mode (default), (B) LF mode (without alpha-spheres generation), (C) LB mode (default), and (D) LB mode (without alpha-spheres generation). Cartoon and stick models denote protein and ligand structures. The black and red spheres in (A) and (B) show before and after P2C. Colorful spheres in (C) and (D) show clustered unoccupied sites. SiteFinder default settings are used for the generation of alpha-spheres. Note that the parameters of alpha-spheres elimination in (B) and (D) do not optimize.

Two ligands that bind to β-ketoacyl-acyl carrier protein synthase Ш (4Z8D and 5BNS) were aligned with the protein structure alignment (McKinney *et al.*, 2016). Notably, the unoccupied sites were predicted by the default LB mode based on 4Z8D overlap with the ligand of 5BNS (Fig. S8a). Whereas the unoccupied sites of alpha-spheres generated by SiteFinder do not overlap with the ligand in 5BNS (Fig. S8b). The difference in results depends on whether the parameter in the P2C process is optimized. Therefore, it is necessary to re-optimize parameters if P2C is performed using alpha-spheres from other methods.


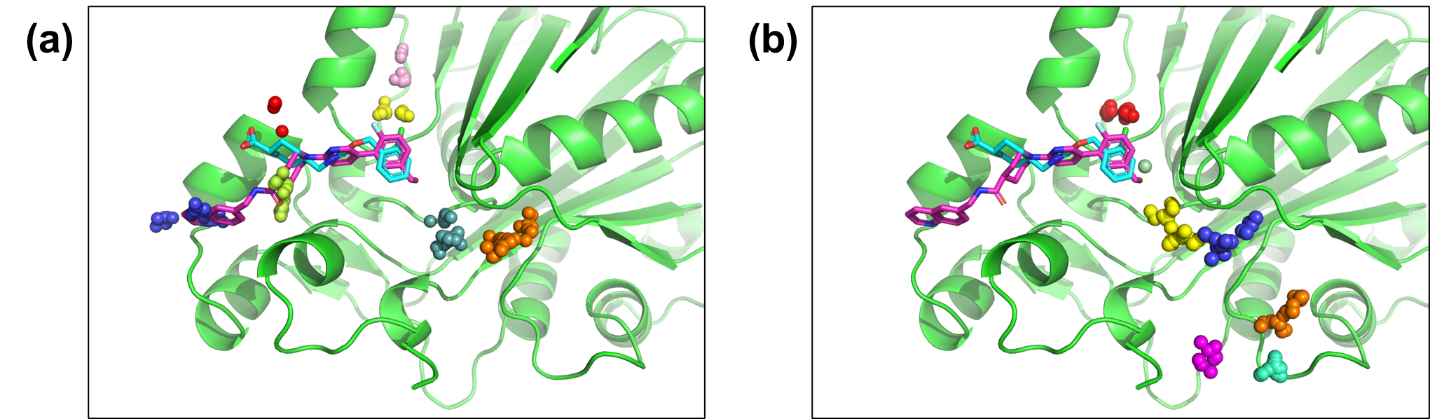
Figure S8. Overlap with the unoccupied sites and another ligand. Cartoon models represent protein structures. Cyan and magenta stick model show the original ligand and another inhibitor that binds in the same protein, respectively. Colorful spheres show the unoccupied sites obtained from (a) the default LB mode and (B) the LB mode of alpha-spheres generated by SiteFinder.

**References**

Aggarwal,R. *et al.* (2021) DeepPocket: Ligand Binding Site Detection and Segmentation using 3D Convolutional Neural Networks. *J Chem Inf Model*.

Berman,H.M. *et al.* (2000) The Protein Data Bank.

le Guilloux,V. *et al.* (2009) Fpocket: An open source platform for ligand pocket detection. *BMC Bioinformatics*, **10**.

Gupta,K. *et al.* (2004) The 2.0 Å Resolution Crystal Structure of Prostaglandin H 2 Synthase-1: Structural Insights into an Unusual Peroxidase. *J Mol Biol*, **335**, 503–518.

Hartshorn,M.J. *et al.* (2007) Diverse, high-quality test set for the validation of protein-ligand docking performance. *J Med Chem*, **50**, 726–741.

Howard,N. *et al.* (2006) Application of fragment screening and fragment linking to the discovery of novel thrombin inhibitors. *J Med Chem*, **49**, 1346–1355.

McKinney,D.C. *et al.* (2016) Antibacterial FabH Inhibitors with Mode of Action Validated in Haemophilus influenzae by in Vitro Resistance Mutation Mapping. *ACS Infect Dis*, **2**, 456–464.

McVey,C.E. *et al.* (2001) Crystal structures of penicillin acylase enzyme-substrate complexes: Structural insights into the catalytic mechanism. *J Mol Biol*, **313**, 139–150.

Oltersdorf,T. *et al.* (2005) An inhibitor of Bcl-2 family proteins induces regression of solid tumours. *Nature*, **435**, 677–681.

Orita,M. *et al.* (2009) Two ‘Golden Ratio’ indices in fragment-based drug discovery. *Drug Discov Today*, **14**, 321–328.

Orth,P. *et al.* (2004) Crystal structure of the catalytic domain of human ADAM33. *J Mol Biol*, **335**, 129–137.

Rasmussen,H.B. *et al.* (2003) Crystal structure of human dipeptidyl peptidase IV/CD26 in complex with a substrate analog. *Nat Struct Biol*, **10**, 19–25.

Su,M. *et al.* (2019) Comparative Assessment of Scoring Functions: The CASF-2016 Update. *J Chem Inf Model*, **59**, 895–913.

Timm,D.E. *et al.* (2001) Crystal structure of thiamin pyrophosphokinase. *J Mol Biol*, **310**, 195–204.

Wunberg,T. *et al.* (2006) Improving the hit-to-lead process: Data-driven assessment of drug-like and lead-like screening hits. *Drug Discov Today*, **11**, 175–180.
